# Metabolism-based artificial organelles: From precise construction to smart theranostics

**DOI:** 10.1016/j.mtbio.2025.102573

**Published:** 2025-11-24

**Authors:** Keqiang Deng, Yihang Zhang, Wenyu Jiang, Yuxin Duan, Mei Gao, Min Zeng, Jiehao Chen, Xiaoting Chen, Zhen Fan, Chengli Yang, Kai Zhou

**Affiliations:** a West China School of Medicine, Sichuan University, 610040, Chengdu, PR China; b Animal Experiment Center, West China Hospital/West China School of Medicine, Sichuan University, 610040, Chengdu, PR China; c Department of Geriatrics, Sichuan Provincial People’s Hospital, University of Electronic Science and Technology of China, Chengdu, Sichuan, PR China; d Department of Pharmacy, Clinical Research Center of Integrated Traditional Chinese and Western Medicine, The Affiliated Hospital of Guizhou Medical University, Guiyang, 550004, PR China; e Sports Medicine Center, West China Hospital, Sichuan University, China/Department of Orthopedics and Orthopedic Research Institute, Chengdu, 610041, PR China

**Keywords:** Artificial organelles, Metabolic engineering, Bottom-up construction, Subcellular targeting, Precision theranostics

## Abstract

Cellular metabolic dysregulation is a core driver of major diseases, including cancer, neurodegenerative disorders, and cardiovascular conditions. However, conventional interventions such as small-molecule drugs and gene editing are hampered by off-target effects, delivery challenges, and a lack of spatiotemporal precision, resulting in a failure to effectively reengineer pathological metabolic networks. Artificial organelles, constructed via a “bottom-up” bioinspired approach, represent a paradigm shift from systemic intervention to “metabolic system reconstruction,” offering a revolutionary strategy to precisely mimic, repair, or augment specific metabolic functions at the subcellular level. Here, we systematically review recent advances in metabolism-based artificial organelles, from their precise construction to smart theranostic applications. We first elaborate on the core construction strategies, including lipid and protein self-assembly, microfluidics, 3D bioprinting and biomembrane fusion for enabling dynamic interactions and content delivery, followed by a dissection of the design principles for modulating three metabolic pillars: (1) remodeling energy metabolism by mimicking mitochondrial function and regulating glycolysis; (2) controlling biosynthesis by emulating the endoplasmic reticulum (ER) and substance transport networks; and (3) reshaping redox homeostasis by mimicking peroxisomes through multienzyme cascades and intelligent responsive systems that precisely regulate signaling molecules such as reactive oxygen species (ROS). We link these functional designs to specific metabolic vulnerabilities in diseases and showcase applications in neurodegenerative disorders, cancer, cardiovascular diseases, and inflammatory conditions. Specific strategies include repairing damaged neurons through synergistic energy supplementation and antioxidation or inhibiting tumors via a combination of “starvation therapy” and pro-oxidative “gas therapy.” Finally, we critically address the key challenges in biocompatibility, systemic complexity, *in vivo* delivery, and clinical translation and outline future perspectives toward intelligent, autonomous systems integrated with artificial intelligence (AI)-driven design and multiscale, “artificial tissue” constructs. This review aims to provide a theoretical framework and technological roadmap for designing the next generation of smart metabolic intervention tools.

## Graphical abstract

**Scheme**. Artificial organelles (AOs) represent a paradigm shift from systemic intervention to ‘metabolic system reconstruction’ for treating diseases driven by metabolic dysregulation. This review presents a comprehensive framework spanning bottom-up construction strategies (self-assembly to 3D bioprinting), functional designs targeting three metabolic pillars (energy metabolism, biosynthesis, redox homeostasis), and precision therapeutic applications in cancer, neurodegeneration, and inflammatory diseases. We critically analyze translational challenges including biocompatibility, scalability, and *in vivo* delivery, while proposing future directions toward AI-driven intelligent systems. This work provides a roadmap for engineering next-generation metabolic intervention tools.

**Figure:**
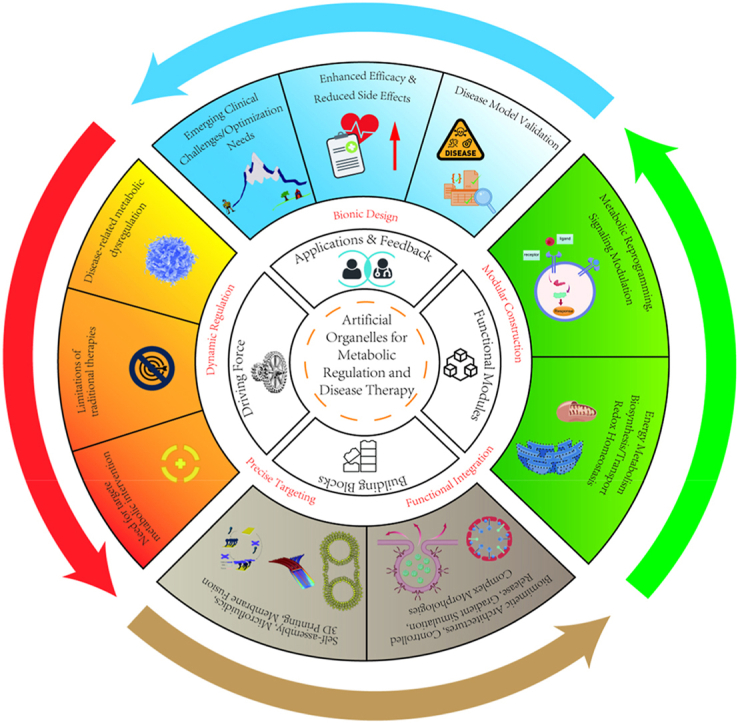

## 1 Introduction

### 1.1 Background

Cellular metabolic regulation serves as the cornerstone of life, dynamically balancing energy supply, material synthesis, and redox homeostasis to maintain cellular survival and function. Dysregulation of these intricate metabolic networks is not merely a symptom but a fundamental driver in the pathogenesis of numerous major diseases, including cancer, neurodegenerative disorders, and cardiovascular conditions. For instance, the Warburg effect reflects a metabolic shift in tumor cells toward hyperactive glycolysis to support rapid proliferation, while mitochondrial dysfunction represents an early event triggering neuronal death in neurodegeneration. Thus, achieving precise modulation of metabolic networks is paramount not only for advancing our fundamental understanding of disease mechanisms but also for developing next-generation targeted therapies [1].

However, conventional metabolic interventions, such as small-molecule inhibitors or gene editing, face considerable clinical limitations. Small-molecule drugs often suffer from significant off-target effects and systemic toxicity, whereas gene-editing technologies, despite their precision, are associated with issues of delivery efficiency and potential immunogenicity [2]. Crucially, both approaches lack the necessary spatiotemporal resolution to act specifically within the diseased subcellular microenvironment, failing to replicate the dynamic and compartmentalized nature of native cellular processes. In this context, artificial organelles have emerged as revolutionary metabolic engineering tools. Built from the ground up using principles of synthetic biology and materials science, these bioinspired, modular constructs are designed to precisely reconstruct or augment specific metabolic functions at the subcellular scale. Lipid self-assembled mitochondrial mimics, for example, have been shown to restore ATP synthesis with remarkable efficiency [3], whereas microfluidically engineered multilayered vesicles can replicate the complex transport logistics of the endoplasmic reticulum–Golgi network [4]. Such breakthroughs represent a paradigm shift from broad, systemic pathway intervention to a more sophisticated strategy of “metabolic system reconstruction,” demonstrating transformative potential in regenerative medicine, precision oncology, and immune modulation.

### 1.2 Overview and historical progression of artificial organelles

Artificial organelles are defined as engineered nanostructures or microstructures capable of emulating the core functions of natural organelles (*e.g.*, catalysis, transport, or signaling) and programming biochemical reactions within intra- or extracellular environments [5,6]. Their design integrates interdisciplinary advances in synthetic biology, materials science, and nanotechnology, aiming to reconstruct functional modules of living systems through a “bottom-up” synthetic strategy [6]. At the very beginning, a critical distinction must be made between metabolism-based artificial organelles (AOs) and conventional nanocarriers or nanozymes [7,8]. While many nanoplatforms utilize similar fabrication techniques like self-assembly, they often serve as simple delivery vehicles or single-catalyst systems [9,10]. In contrast, ‘true’ AOs, as defined in this review, embody a higher level of biomimicry and functional complexity. They should possess at least two of the following three core traits: (1) a well-defined compartmentalized structure creating a distinct internal microenvironment, mimicking natural organelle boundaries [11,12]; (2) integrated multi-enzyme cascades or metabolite transport capabilities that reconstruct metabolic pathway segments, rather than single reactions [13]; and (3) capacity for dynamic interaction with host cell metabolic networks, enabling subcellular localization, feedback regulation, or inter-organelle communication [7]. This ‘systems reconstruction’ approach elevates AOs beyond traditional nanomedicine.

The development of artificial organelles has progressed through distinct phases. Early efforts focused on simple lipid vesicles or protein cages to mimic basic membrane compartmentalization [14]. Subsequent advancements leveraged microfluidics and 3D printing to achieve uniform sizing and the integration of simple enzyme cascades [[15], [16], [17], [18]]. The current era is characterized by a drive toward “intelligent” and “dynamic” systems [19,20]. This progress is fuelled by deeper insights into nonequilibrium self-assembly [21], the development of stimuli-responsive materials (*e.g.*, smart hydrogels), and the sophisticated engineering of biomimetic communication. For example, light-controlled artificial peroxisomes can now dynamically modulate cellular signaling via precise ROS regulation [22], while SNARE protein-integrated systems facilitate biomimetic vesicle targeting and fusion, replicating intercellular communication with high fidelity [23,24]. These milestones not only deepen our understanding of biological self-organization but also lay the technical groundwork for creating therapeutic “cell-mimetic machines.”

A diverse toolbox of construction strategies underpins this field: Lipid and protein self-assembly remains the gold standard for replicating the dynamic and flexible nature of organelle membranes [25,26]. Microfluidic technology enables high-throughput fabrication and the creation of complex gradients, which are essential for mimicking the cellular microenvironment [27]. Moreover, 3D bioprinting overcomes the challenge of recreating complex, multiscale architectures, opening new avenues for tissue-level metabolic engineering [28]. Critically, the convergence of these techniques with advanced analytical tools such as multiomics and single-cell analysis is enabling a transition from *in vitro* models to *in vivo* integrated systems, heralding a new frontier in personalized and precision medicine.

## 2 Construction strategies for artificial organelles

To realize the ambitious goal of reconstructing metabolic modules from the ground up, a sophisticated and versatile toolbox of construction strategies is essential. These strategies are guided by the core principles of biomimicry, modularity, programmability, and dynamic responsiveness. This chapter details the primary technological pillars—self-assembly, microfluidics, 3D bioprinting, and biomembrane fusion—and critically evaluates their unique contributions and inherent limitations. We explore how these methods provide the fundamental building blocks for creating structures that range from simple enzyme-loaded vesicles to complex, multicompartment systems, thereby laying the physical groundwork for the functional designs discussed in the subsequent chapter.

### 2.1 Self-assembly technology

Self-assembly is the cornerstone of artificial organelle construction, leveraging intrinsic molecular interactions to spontaneously form ordered, biomimetic structures, ultimately achieving dynamic assembly and recognition at the molecular level.

#### 2.1.1 Lipid self-assembly: crafting the biomimetic boundary

Lipid molecules, the primary components of cell membranes, spontaneously self-assemble into stable bilayer vesicles (liposomes) in aqueous environments because of their amphiphilic nature. This process, driven primarily by hydrophobic interactions, creates a selectively permeable barrier that establishes the essential compartmentalization of an organelle [25]. The physicochemical properties of the resulting membrane—such as fluidity, thickness, and charge—are highly programmable by tuning the lipid composition (*e.g.*, acyl chain length, saturation), temperature, and ionic strength [29,30]. For example, the inclusion of unsaturated lipids increases membrane fluidity, which is crucial for dynamic processes, whereas charged lipids can influence protein interactions and stability [26]. Furthermore, adjusting the ionic strength and temperature can achieve reversible polymerization and dispersion of liposomes, which is highly important for applications such as drug delivery and bioimaging [31]. While lipid self-assembly offers unparalleled biomimicry and flexibility, achieving robust long-term stability in complex biological fluids and ensuring precise control over the encapsulation efficiency of diverse biomolecules remain significant challenges. The groundbreaking work by Song et al. demonstrating how self-organizing microcapsules can evolve to mimic cellular functions underscores the potential of this approach [2].

#### 2.1.2 Protein self-assembly: engineering the functional core

Protein self-assembly enables the creation of highly defined, functional nanostructures, such as nanocages and filaments [32]. The assembly process is encoded within the protein’s primary sequence, which dictates its folding into specific secondary (α-helices, β-sheets) and tertiary structures. Under appropriate conditions (pH, ionic strength, and temperature), these protein monomers can spontaneously organize into higher-order architectures [33]. Ferritin, a natural iron-storage protein, is a classic example of a 24-subunit nanocage that has been extensively repurposed as a nanoreactor and delivery vehicle [2]. The key advantage of protein-based assembly is the genetic programmability of its building blocks, allowing for precise engineering of functionalities through mutation and fusion [34]. However, the production of complex recombinant proteins can be challenging, and off-pathway aggregation is a common issue that can compromise yield and function [35].

#### 2.1.3 Hierarchical assembly and functional emergence in multiprotein systems

The assembly of multiprotein systems introduces a higher level of complexity, where hierarchical regulation and emergent functions become central themes. By combining different proteins, researchers can create structures with capabilities exceeding the sum of their parts. This hierarchical control is achieved by programming specific, sequential protein‒protein interactions, which are often modulated by environmental cues or enzymatic activity. For example, Ge et al. designed neuropeptide-based artificial aggregates capable of complex signaling cascades, demonstrating how interactions between components give rise to new biological functions [36]. A key scientific challenge in this area is to decipher the “design rules” that govern how function emerges from multicomponent interactions, moving from trial-and-error to rational, predictable design. Recent advances in the use of DNA nanostructures as programmable scaffolds for organizing protein arrays offer a powerful route to address this challenge [37].

In essence, self-assembly provides the foundational ‘chassis’ and ‘boundary’ for AOs, offering unparalleled biomimicry. Its primary strength lies in creating dynamic interfaces capable of responding to environmental cues, while its main challenges are batch-to-batch reproducibility and long-term stability *in vivo*—limitations that complementary techniques like microfluidics aim to address.

### 2.2 Microfluidic technology: precision engineering of micro-compartments

Microfluidics provides unparalleled precision in manipulating fluids at the micrometer scale, enabling the high-throughput production of artificial organelles with exceptional control over size, structure, and composition. Traditional microfluidics includes channel-based and digital systems, with paper-based microfluidics increasingly used in recent years [38,39]. Paper-based devices typically handle monophasic flows, whereas channel and digital systems can process both monophasic and oil–aqueous biphasic flows. Each approach has its own advantages and limitations, so the choice depends on the specific application [38].

#### 2.2.1 Principles of microfluidic fabrication

Microfluidic devices utilize precisely engineered microchannels to control fluid flow, where laminar flow dominates and mixing occurs primarily through diffusion. By manipulating the channel geometry (*e.g.*, flow-focusing or T-junctions) and the flow rates of immiscible fluids, highly monodisperse droplets can be generated [40]. These droplets serve as templates for forming vesicles and other compartments. The key advantage is reproducibility, overcoming the polydispersity issues common in bulk methods. The surface properties of the channels (hydrophilicity/hydrophobicity) are also critical for controlling droplet formation and stability [4].

#### 2.2.2 Advanced structures and gradient simulation

Microfluidics excels in fabricating advanced structures that are difficult to achieve with other methods. By using multiphase flows (*e.g.*, water-in-oil-in-water double emulsions), it is possible to construct multilayered vesicles (vesosomes) that mimic the nested structure of organelles such as mitochondria. This layering allows for the encapsulation of different components in distinct compartments, facilitating multistep enzymatic reactions [41].

Furthermore, microfluidic systems are uniquely suited for generating and maintaining stable chemical gradients, which are fundamental to many cellular processes. By controlling the diffusion of molecules from parallel streams, researchers can create microenvironments with defined concentration profiles, simulating the signaling gradients that cells experience *in vivo*. This capability is not merely for creating multilayered structures; it is the core principle that allows for the dynamic study of processes such as chemotaxis, cell‒cell communication, and spatially organized metabolic pathways within artificial cells [27]. The integration of these two aspects—structural complexity and gradient generation—represents the true power of microfluidics in this field.

**Fig. 1:**
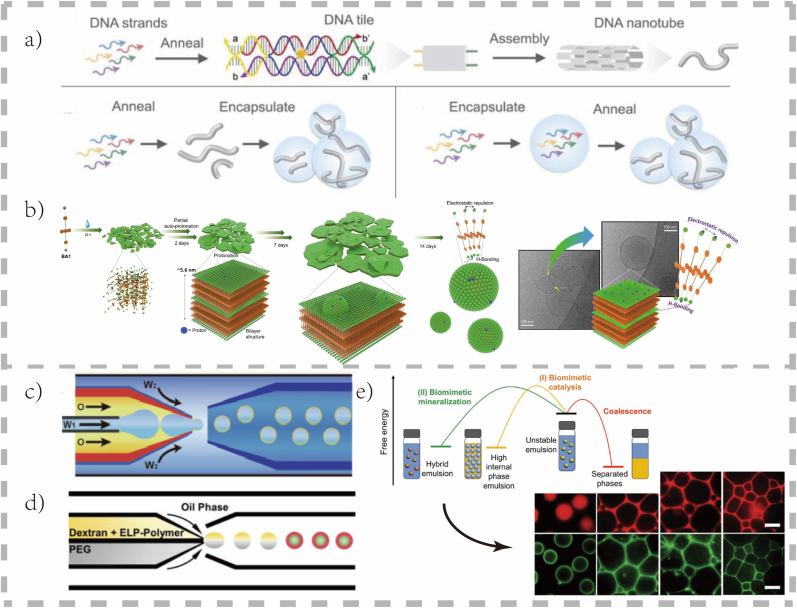
**Fundamental Assembly Strategies (Self-Assembly and Microfluidics)**. a) Artificial cytoskeleton: Schematic of DNA tiles and nanotubes and different methods for encapsulating and self-assembling DNA nanotubes from preformed monomers at constant temperature [29].© 2021, Nat Commun. b) Artificial vesicle: Schematic illustration of spontaneous curvature induction in a self-assembled bilayer membrane. The autogenous partial protonation of the tertiary amine units and the resulting asymmetric membrane led to the formation of vesicles [30].© 2024, Angew Chem Int Ed Engl. c) Artificial vesicle: Schematic illustration and optical micrograph showing the production of Pluronic-based polymersomes via a glass capillary-based microfluidic device using water-in-oil-in-water (W1/O/W2) double emulsion droplets as templates. The scale bar represents 100 μm [40].© 2022, Nat Commun. d) Artificial vesicle: Illustration of the microfluidic preparation of wate‐in‐oil emulsion‐based protocellular entities, in which ELP‐b‐Dex or ELP‐b‐PEG is individually encapsulated together with the dextran/PEG system; here, the upper phase is dextran and ELP‐polymer conjugates, and the lower phase is PEG [27].© 2021, Adv Sci (Weinh). e) Artificial metabolic compartment: A Pickering emulsion can convert substrates into organic or inorganic products, inducing complex self-organization and structuring of cell-sized microcompartments [2].© 2025,Small.

The power of microfluidics lies in its high-throughput precision for creating uniform artificial organelles and compartments. Its key capabilities include engineering sophisticated, multi-layered structures (*e.g.*, vesosomes) and simulating dynamic environments through stable chemical gradients, uniquely integrating structural and environmental control for advanced cell research. The representative construction Strategies mentioned in Sec. 2.1 and 2.2 are detailed in Fig. 1.

### 2.3 3D bioprinting: constructing multi-scale architectures

3D bioprinting extends the principles of additive manufacturing to biological systems, enabling the construction of complex, three-dimensional structures with microscale resolution, bridging the gap between individual organelles and tissue-level organization.

#### 2.3.1 Principles of material selection and printing

The successful 3D bioprinting of artificial organelle-laden constructs relies on “bioinks” that meet stringent criteria: biocompatibility, printability (rheological properties), and functional relevance. Materials such as gelatin methacrylamide (GelMA) and poly(lactic-co-glycolic acid) (PLGA) are popular choices because of their tunable mechanical properties and biocompatibility [42]. A critical consideration is that the printing process itself (*e.g.*, shear stress in extrusion printing, light exposure in photopolymerization) must not compromise the integrity or activity of the embedded artificial organelles. Optimizing printing parameters—nozzle diameter, speed, and pressure—is crucial for achieving high fidelity and cell viability [28].

#### 2.3.2 Creating functional complexity and hierarchical structures

The primary advantage of 3D bioprinting is its ability to create spatially organized, heterogeneous structures. By using multimaterial printing, different types of artificial organelles can be deposited at specific locations within a larger scaffold, mimicking the functional zonation of tissues. For example, one could print a structure with “mitochondria-rich” zones for high energy demand and “ER-rich” zones for protein synthesis. This technology is not just about recreating static shapes; it is about engineering functional microtissues where the spatial arrangement of artificial organelles dictates the overall metabolic behavior [43,44]. The integration of sacrificial support materials allows for the printing of intricate internal networks, such as perfusable channels that mimic vascular networks, which are essential for nutrient supply and waste removal in larger constructs [45].

3D bioprinting creates complex 3D biological structures using specialized “bioinks” that are biocompatible and preserve the function of embedded components. Its key advantage is the precise spatial arrangement of these components to build functional, hierarchical tissues. This allows for the creation of distinct functional zones and integrated vascular networks, engineering active microtissues that go beyond simple structural replication.

### 2.4 Biomembrane fusion: enabling dynamic interaction and delivery

Biomembrane fusion is a critical biological process that enables communication and material exchange between organelles. Engineering this process into artificial systems unlocks dynamic functionalities such as targeted delivery and content mixing.

#### 2.4.1 Molecular machinery and control

The molecular basis of controlled membrane fusion is often inspired by the natural soluble N-ethylmaleimide-sensitive factor attachment protein receptor (SNARE) protein complex. By incorporating complementary v-SNAREs and t-SNAREs onto the membranes of different vesicle populations, researchers can program specific fusion events [23]. As discussed in Section 2.1.1, the lipid composition of the interacting membranes plays a crucial modulatory role, affecting membrane curvature and fluidity, which are critical for overcoming the energy barrier to fusion [24]. The kinetics of fusion can be quantitatively analyzed via techniques such as time-resolved fluorescence microscopy, providing insights into the influence of protein concentration, lipid type, and temperature [26]. Other triggers, such as light or changes in pH, can also be engineered to control fusion events spatiotemporally.

#### 2.4.2 Applications in constructing advanced organelles

Biomembrane fusion technology is a powerful tool for constructing complex, functional artificial organelles postfabrication. It allows for the targeted delivery of “cargo” (*e.g.*, enzymes, genes) from one vesicle population to a “reactor” vesicle, initiating a reaction upon fusion. This strategy is particularly valuable for handling sensitive components that cannot withstand initial fabrication conditions. Furthermore, it enables the creation of dynamic systems where artificial organelles can interact with and deliver their contents directly into living cells by fusing with the plasma membrane, a key step toward *in vivo* therapeutic applications [46].

**Fig. 2:**
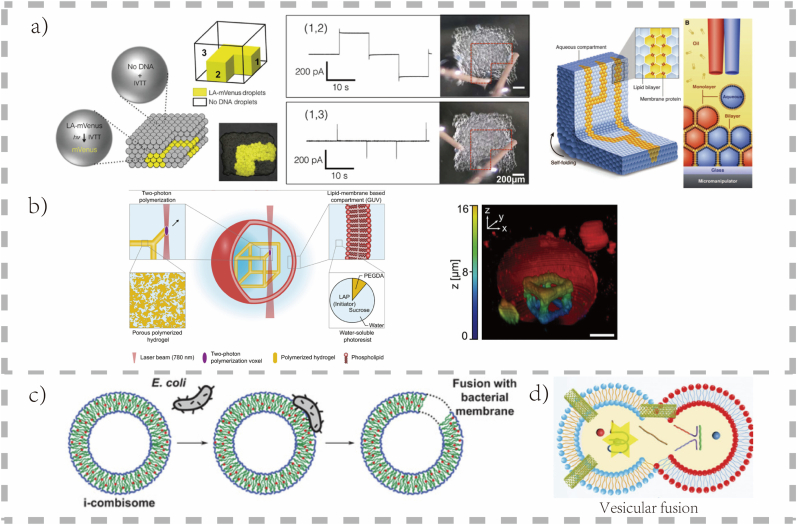
**Advanced Fabrication (3D Bioprinting and Membrane Fusion)**. a) Directional electrical communication in 3D-printed synthetic tissue induced by activated patterned expression of pore proteins. Scheme and electrical recordings show signal detection between electrodes at positions 1 and 2, but not at l and 3 [47].©2016, AAAs. Scheme of a printed droplet network, which involves a printing process in which two droplet generators eject droplets [48].©2013, Science. b) 3D laser printing in synthetic cells. Schematic illustration of a 3D hydrogel cube frame (yellow) as an exemplary object that was written into a preformed giant unilamellar lipid vesicle (GUV, red) by two-photon polymerization [42].© 2022, Adv Mater. c) Fusion-based hybrid system of i‐combisomes and bacteria, enabling chemical and biological surface design within a single protocell. This approach allows direct capture of bacterial membrane active receptors without purification [26].© 2022, Adv Sci (Weinh). d) The CNT-embedded artificial cell (ArtifCell@CNT) model effectively promoted cell‒cell fusion compared with the model without CNTs [46].© 2025, Adv Mater. (For interpretation of the references to colour in this figure legend, the reader is referred to the Web version of this article.)

Engineered biomembrane fusion creates dynamic artificial systems that communicate and exchange materials. By mimicking natural proteins and using triggers like light, programmable fusion is achieved. This enables two major functions: the modular assembly of complex organelles and direct therapeutic delivery to living cells. This technology advances static constructs into interactive systems capable of on-demand functions. The representative construction strategies mentioned in Sec. 2.3 and 2.4 are detailed in Fig. 2.

### 2.5 Synergistic integration of construction strategies

While each construction strategy offers unique advantages, the future of the field lies in their synergistic integration. Advanced artificial organelles are not built via a single method but rather via a multistep process that combines the strengths of different techniques [49]. For example, 1) microfluidics-assisted self-assembly involves the use of microfluidics to precisely control the conditions (*e.g.*, concentration gradients, mixing times) under which lipids and proteins self-assemble, leading to higher-order structures with superior monodispersity and complexity [50]. 2) 3D printing of functionalized vesicles: Synthesizing functionally distinct populations of vesicles via self-assembly or microfluidics and then using 3D bioprinting to organize them spatially into a larger, structured “meta-organelle” or artificial tissue.

This integrated approach is essential for bridging the scales from molecular assembly to macroscopic function, paving the way for the creation of truly lifelike and therapeutically potent artificial organelle systems.

### 2.6 Critical comparison of construction strategies

The construction strategies detailed in this chapter—self-assembly, microfluidics, 3D bioprinting, and biomembrane fusion—each provide a unique pathway to engineer artificial organelles. A critical comparison reveals that no single method is universally superior; rather, the choice of strategy is a trade-off dictated by the specific application requirements, particularly concerning structural fidelity, functional complexity, scalability, and biocompatibility. This section evaluates these core technologies against these critical benchmarks. The summary is shown in Table 1.

**Table 1:** Critical comparison of artificial organelle construction strategies.

Construction Strategy	Key Strengths	Key Limitations	Key Challenges
Self-Assembly (Lipid/Protein)	1) High biomimicry; 2) Bottom-up formation; 3) Inherent scalability for basic structures; 4) High flexibility in composition.	1) Spontaneous & stochastic process; 2) Low precise control; 3) Polydisperse populations; 4) Difficulty with multi-component systems.	Scalability: Excellent for mass production of simple vesicles. Immunogenicity: Low for lipids; High risk for recombinant proteins and aggregates.
Microfluidics	1) High precision & monodispersity; 2) Complex structures (*e.g.*, vesosomes); 3) Gradient generation; 4) High-throughput.	1) Operational complexity; 2) Device fabrication; 3) Risk of channel clogging; 4) Potential shear stress on biomolecules.	Scalability: High-throughput but limited to chip-based systems, industrial scale requires complex parallelization. Immunogenicity: Dependent on encapsulated materials, process itself is low risk.
3D Bioprinting	1) Spatially defined, hierarchical architectures; 2) Functional zonation; 3) Bridges organelle-to-tissue scale.	1) Resolution-viability trade-off; 2) Slow production speed; 3) Potential damage from printing process (shear, UV).	Scalability: Suitable for macroscopic constructs, but slow for producing basic building blocks. Immunogenicity: Risk from bioink materials and from biomolecule damage during printing.
Biomembrane Fusion	1) Dynamic interactivity & communication; 2) Temporal control; 3) Targeted delivery; 4) Bio-orthogonal functionality.	1) Low fusion specificity & yield; 2) Off-target fusion; 3) Not a primary construction method.	Scalability: Low, typically used for specific, targeted fusion events rather than mass production. Immunogenicity: Risk from exogenous fusion machinery (*e.g.*, engineered peptides/proteins).

#### 2.6.1 Self-assembly: biomimicry at the cost of control

Self-assembly remains the foundational strategy, leveraging intrinsic molecular interactions to form structures from the bottom-up. Its principal advantage is its high biomimicry, producing structures like liposomes and protein cages that closely resemble their biological counterparts [25,28]. This approach is inherently scalable for mass production of basic vesicular structures, as it often involves simple mixing in bulk solutions. However, this scalability comes at the expense of precise control, typically resulting in polydisperse populations that are unsuitable for applications requiring uniformity [40]. A major limitation is the spontaneous and stochastic nature of the process, making it difficult to orchestrate the formation of complex, multi-component systems with high spatial fidelity. Furthermore, while lipid-based assemblies generally exhibit low immunogenicity, protein-based self-assembly carries a higher risk. Recombinant or engineered proteins can elicit unintended immune responses, and the common issue of off-pathway aggregation can generate immunogenic protein aggregates [35]. Therefore, while self-assembly is unparalleled for creating basic, scalable, and highly biomimetic compartments, its limitations in precision and potential immunogenicity constrain its use in sophisticated or therapeutic applications.

#### 2.6.2 Microfluidics: precision and throughput with operational complexity

Microfluidics addresses the critical limitation of self-assembly by providing unparalleled precision and monodispersity in the production of artificial organelles [40]. Its ability to generate complex, multilayered architectures (*e.g.*, vesosomes) and stable chemical gradients enables the engineering of advanced microenvironments for studying compartmentalized reactions [27,41]. This strategy offers a favourable balance, enabling high-throughput generation of highly uniform compartments. However, its scalability is paradoxical; while it can produce vast numbers of droplets, the process is typically confined to chip-based systems, and scaling to industrial volumes often requires parallelization of numerous devices, increasing complexity and cost. The immunogenicity of the final product is largely determined by the materials used (*e.g.*, lipids, polymers), but the process itself is inert. The primary limitations are operational, including the sophistication of device fabrication, susceptibility to channel clogging, and the potential for shear stress to damage sensitive biomolecules during encapsulation.

#### 2.6.3 3D bioprinting: architectural mastery at the macroscale

3D bioprinting’s unique contribution is its ability to create spatially defined, multi-scale architectures, bridging the gap from individual organelles to functional tissues [43,45]. Its strength lies in the deliberate programming of structural hierarchy, allowing for the creation of “meta-organelles” with functional zonation. In terms of scalability, bioprinting is well-suited for creating macroscopic tissue constructs for *in vitro* modeling or regenerative medicine, but the process is inherently slower than microfluidic droplet generation for producing the basic organelle building blocks. The challenges of immunogenicity are multifaceted. While the bioinks (*e.g.*, GelMA, PLGA) are chosen for biocompatibility [42], the printing process itself—through shear stress or UV exposure—can compromise the integrity of encapsulated biologicals, potentially increasing their immunogenic potential [28]. The primary limitation is the resolution-viability trade-off; achieving the fine resolution needed to print subcellular structures often requires conditions (*e.g.*, high pressure, small nozzles) that are detrimental to the viability and function of the encapsulated artificial organelles.

#### 2.6.4 Biomembrane fusion: dynamic interactivity with specificity challenges

Biomembrane fusion is less a construction strategy and more a dynamic functionalization tool. It enables post-assembly integration and communication, allowing for the creation of complex systems through targeted cargo delivery and content mixing [23,46]. Its key advantage is the introduction of temporal control and bio-orthogonal interactivity, both between artificial compartments and with living cells. The scalability of this approach is low, as it is typically used to fuse specific, pre-formed vesicle populations and is not a mass-production technique. The critical challenge lies in achieving high specificity and yield. While SNARE-protein mimics offer a biological mechanism, achieving fusion efficiencies comparable to natural systems is difficult, and off-target fusion remains a concern [24,26]. The immunogenicity risk is directly tied to the fusion machinery; the introduction of exogenous peptides or proteins (*e.g.*, engineered SNAREs) can provoke an immune response if deployed *in vivo*.

#### 2.6.5 Towards a synergistic paradigm

The critical comparison underscores that the future of artificial organelle construction lies not in a single, dominant technology but in the synergistic integration of these strategies [49,50]. For instance, the high-throughput precision of microfluidics can be used to produce monodisperse, functionalized vesicles, which are then spatially organized into a larger, hierarchical architecture via 3D bioprinting. Simultaneously, the dynamic functionality of these printed structures can be updated or activated on-demand through biomembrane fusion events. This integrated approach allows researchers to mitigate the inherent limitations of each standalone method, paving the way for the creation of complex, robust, and clinically relevant artificial organelle systems that truly mirror the sophistication of their natural counterparts.

## 3 Core functional design for metabolic regulation

The construction strategies detailed in Chapter 2 provide the physical scaffolds and compartmentalization necessary for recreating subcellular architectures. The ultimate therapeutic value of these artificial organelles, however, lies in their ability to execute specific, preprogrammed metabolic functions. This chapter delves into the core functional designs tailored to modulate the three pillars of cellular metabolism: energy metabolism, substance biosynthesis and transport, and redox homeostasis. Moving beyond simple encapsulation, the focus here is on the rational engineering of enzymatic cascades, transport systems, and signaling circuits. We will illustrate how the choice of a construction method (*e.g.*, self-assembly for dynamic membranes or microfluidics for multistep reactions) is intrinsically linked to achieving a desired metabolic outcome, thereby laying the groundwork for targeting the specific metabolic vulnerabilities of major diseases. The tightness of the connection between artificial organelle construction strategies and their metabolic functions is detailed in Table 2.

**Table 2:** Artificial organelle construction—metabolic function connectivity matrix.

Construction Strategy	Metabolic Function Category	Star Rating	*In Vivo* Verified	Scalable	Clinical Potential	Rationale/Key Features
	Energy Metabolism Regulation					
Self-Assembly (Lipid/Protein)		★★★★	✓	✓	High	Lipid-encapsulated respiratory chain complexes have high functional biomimicry; protein nanocages for glycolytic enzymes are well-verified
Microfluidics		★★★★	✓	✓	Medium	Gradient-controlled glycolytic flux mimics physiological metabolism; multilayered vesicles for ATP synthesis are verified *in vitro* and *in vivo*
3D Bioprinting		★★☆	✗	✗	Medium	Vascularized oxygen-diffusion scaffolds have moderate functional complexity; shear stress during printing may damage delicate enzyme complexes, and only small-scale *in vitro* verification is available
Biomembrane Fusion		★★★☆	✓	✓	Medium	Mitochondrion-targeted ATP delivery systems have relatively high functional biomimicry
	Substance Biosynthesis & Transport					
Self-Assembly (Lipid/Protein)		★★★★	✓	✓	High	Liposome-based ER protein folding mimics are functionally complex and biomimetic; ferritin-mediated apolipoprotein transport is well-verified and scalable
Microfluidics		★★★★	✓	✓	High	W/O/W emulsions for ER-Golgi transport have high functional biomimicry; microbead enzyme immobilization technology is scalable and well-verified
3D Bioprinting		★★★★	✗	✗	High	Tissue-engineered lipid synthesis modules are functionally complex; spatially programmed enzyme scaffolds achieve tissue-level spatial organization
Biomembrane Fusion		★★★★	✓	✓	High	SNARE-mediated interorganelle transport has high functional biomimicry; membrane fusion technology is very potential
	Redox Homeostasis Modulation					
Self-Assembly (Lipid/Protein)		★★★★	✓	✓	High	Nanozyme-based peroxisome mimics have high functional biomimicry; redox regulation effect is remarkable
Microfluidics		★★☆	✗	✓	Medium	pH-responsive ROS generators have moderate functional complexity; only small-scale *in vitro* verification is available
3D Bioprinting		★★☆	✗	✗	Medium	Antioxidant hydrogel matrices have moderate functional complexity; only *in vitro* verification is available, and large-scale printing is difficult
Biomembrane Fusion		★★★☆	✓	✓	Medium	Limited by moderate-level functional biomimicry and clinical potential, the rating is lowered.

Star rating weighting: Functional Complexity & Biomimicry (40 %), Manufacturing Maturity & Scalability (30 %), Verification Level (30 %). *In Vivo* Verified (✓/✗): Whether functional verification is completed in *in vivo* environments such as animal models. Scalable (✓/✗): Whether there is potential for batch preparation or large-scale application. Clinical Potential (High/Medium/Low): Expectation of clinical translation based on functional effectiveness, safety, and accessibility. This evaluation synthesizes findings from more than 50 reviewed literatures to ensure comprehensiveness and objectivity of the conclusions.

### 3.1 Functional classification based on organelle mimicry

To translate the core metabolic functions into tangible designs, we adopt a framework inspired by nature’s own subdivisions: organelle mimicry. Classifying artificial organelles (AOs) based on their biological counterparts—such as mitochondria, peroxisomes, endoplasmic reticulum, and lysosomes—provides a structured blueprint for engineering specific metabolic capabilities, as outlined in Table 3. This biomimetic approach ensures that design efforts are grounded in proven biological principles.

**Table 3:** Comparison and chapter outlines of artificial organelle mimics.

Organelle Mimicry	Chapter Outlines	Representative Strategies	Key Challenges	Performance Metrics
Artificial Mitochondria	Sec. 3.2	1) Bottom-Up Construction 2) Mitochondrial Transplantation 3) Semi-Synthetic Systems	1) Homeostatic Control 2) Targeted Delivery 3) Functional Assembly 4) Therapeutic Safety	1) ATP Yield 2) Membrane Potential 3) Functional Rescue 4) Biodistribution
Artificial Peroxisomes/Nanozymes	Sec. 3.4	1) Polymersome Encapsulation 2) Membrane Pores 3) Cell Targeting	1) Permeability-Stability 2) Enzyme Viability 3) Escape-Biocompatibility 4) Scalable Quality	1) Activity Assays 2) Cellular Uptake 3) ROS Reduction 4) Stability Validation
Artificial Endoplasmic Reticulum	Sec. 3.3	1) Proteostasis Support 2) Calcium Regulation 3) Synthetic Inter-Organelle Signaling	1) Environmental Replication 2) Spatiotemporal Control 3) Dynamic Adaptation	1) Refolding Efficiency 2) Calcium Buffering 3) Stress Reduction
Artificial Lysosomes	Sec. 3.3	1) Enzyme Delivery Vehicles 2) Autonomous Degradative Compartments 3) Targeted Clearance Agents	1) Operational Stability 2) Precision Targeting 3) Intracellular Trafficking 4) Manufacturing Complexity	1) Catalytic Efficiency 2) Functional Recovery 3) Targeting Accuracy

The utility of this framework, however, becomes fully apparent when these discrete mimics are deployed to address complex metabolic dysfunctions. Consequently, the following sections will not be constrained by this classification but will instead explore how these biomimetic modules are harnessed to achieve broader metabolic goals. We will examine how artificial mitochondria and glycolytic regulators are applied to remodel energy metabolism (Sec. 3.2), how artificial ER and communication networks regulate substance biosynthesis and transport (Sec. 3.3), and how artificial peroxisomes and redox modulators control redox homeostasis (Sec. 3.4). This shift in perspective, from isolated mimicry to integrated pathway engineering, is essential for developing the sophisticated, multi-task systems envisioned in Section 3.5.

### 3.2 Remodeling energy metabolism

Energy metabolism, which is centered on ATP production and consumption, is the most fundamental process of life. Its dysregulation is a hallmark of numerous pathologies.

#### 3.2.1 Mitochondrial function mimicry: recharging the cellular powerhouse

Artificial mitochondria (or “mitochondrion mimetics”) are designed to supplement or replace dysfunctional native mitochondria. Key strategies include the following: 1) Respiratory chain reconstruction: Coencapsulating components of the electron transport chain (*e.g.*, cytochrome *c*) and ATP synthase into liposomes or polymersomes. For example, Li N. et al. utilized artificial mitochondrial nanobots (AMNs) to encapsulate high-energy phosphate bonds (such as precursors of ATP). These nanobots maintain proton gradients through lipid bilayers, target ischemic cardiomyocytes after oral administration, continuously supply energy for 12 h, and repair energy metabolism [51].The choice of a lipid bilayer (Sec. 2.1.1) is critical here, as it must maintain the proton gradient essential for oxidative phosphorylation [3,51]. 2) Targeted ATP delivery: nanocarriers that encapsulate ATP and release it specifically at sites of high energy demand, such as ischemic tissues or synapses, are designed. Responsiveness can be engineered by using pH-sensitive materials that disassemble in the acidic microenvironment of ischemic tissue, ensuring an on-demand energy supply [52]. 3) Protecting and augmenting native mitochondria: Artificial mitochondrial technology is shifting from “injury substitution” to “functional augmentation” by clearing excess reactive oxygen species (ROS) or delivering key cofactors (*e.g.*, NAD^+^), thereby increasing the self-repair capacity of natural mitochondria. For example, for the targeted delivery of ROS scavengers, Kang et al. developed an artificial mitochondria-targeting peptide (CAMP) that precisely delivers the metallothionein hMT1A to mitochondria in Parkinson’s disease model neurons. This significantly ameliorated excessive ROS, restored tyrosine hydroxylase expression and mitochondrial function, and rescued motor deficits and neuronal degeneration [53].

#### 3.2.2 Glycolysis pathway modulation

Glycolysis is another central energy pathway that is often hyperactivated in cancer (the Warburg effect). Artificial organelles offer precise tools to intervene in this process.

##### 3.2.2.1 Design and optimization of artificial glycolytic regulators

Achieving precise control over glycolysis requires the rational design of artificial organelles that can both house and regulate key enzymes.

This is accomplished through two primary approaches: 1) Enzyme immobilization and cascade assembly: Key glycolytic enzymes (*e.g.*, hexokinase and phosphofructokinase) are immobilized onto scaffolds within an artificial compartment. This spatial organization, often achieved via protein self-assembly (Sec. 2.1.2) or microfluidic templating (Sec. 2.2), mimics the native cytoplasmic “metabolon,” enhancing catalytic efficiency by channeling substrates and minimizing intermediate leakage [54]. 2) Enzyme Engineering for enhanced regulation: The catalytic activity of encapsulated enzymes can be fine-tuned through protein engineering. This includes creating allosteric sites responsive to specific disease biomarkers (*e.g.*, the ATP/ADP ratio) [55] or developing photoswitchable enzymes whose activity can be controlled externally with light [56], offering unparalleled spatiotemporal precision. The latter has achieved millisecond-precision spatiotemporal control in coacervate microreactors [56].

##### 3.2.2.2 Precise regulation of glycolytic flux

The successful integration of these design strategies enables the precise modulation of glycolytic flux. For example, an artificial organelle could be designed to act as a “metabolic sink,” competitively consuming glucose to starve cancer cells. Conversely, under ischemic conditions, another design could facilitate a controlled glycolytic burst to rapidly generate ATP. This demonstrates a shift from simple enzyme inhibition to sophisticated pathway reengineering.

### 3.3 Regulation of substance bioynthesis and transport

Cells rely on a complex network of organelles (ER, Golgi, and lysosomes) for the synthesis, modification, and transport of biomolecules. Artificial systems can mimic and repair these crucial supply chains.

#### 3.3.1 Endoplasmic reticulum (ER) function mimicry

The ER is a central hub for protein folding and lipid synthesis. Artificial ER mimics aim to alleviate ER stress, a common pathological factor. This can be achieved by designing nanovesicles that 1) assist in protein folding, encapsulating molecular chaperones (*e.g.*, Hsp70) [57] and delivering them into cells to aid in the refolding of misfolded proteins. 2) Regulating calcium homeostasis: Calcium sponges or donors act to buffer intracellular Ca^2+^ levels, a key function of the ER [58].

#### 3.3.2 Inter-organelle communication networks

Recreating the dynamic transport network between organelles is a frontier challenge. Using the biomembrane fusion techniques discussed in Section 2.4, researchers can design “feeder” vesicles (mimicking ER export) that specifically fuse with “processor” vesicles (mimicking the Golgi apparatus). This programmed fusion allows for sequential, multistep modifications of encapsulated substrates, emulating the natural protein glycosylation pathway [46]. Beyond simple vesicle fusion, advanced AOs are being designed to mimic sophisticated inter-organelle communication hubs. A prime example is the simulation of ER-mitochondria contact sites (MAMs), critical for Ca^2+^ signaling and lipid exchange [59,60]. Recent work has demonstrated dual AO populations—one mimicking ER with Ca^2+^-binding proteins, another mimicking mitochondria with ATP synthase—that establish functional contact through engineered surface proteins [61,62]. The IP3R-GRP75-VDAC1-MCU axis at MAMs enables quasi-synaptic Ca^2+^ transfer from ER to mitochondria [63,64]. This biomimetic communication enables coordinated Ca^2+^-dependent ATP production, exemplifying true ‘metabolic system reconstruction’ rather than simple component replacement.

#### 3.3.3 Lipid metabolism regulation

Artificial organelles can be designed to modulate lipid synthesis and transport. For example, systems loaded with enzymes that catalyze the breakdown of excess fatty acids or cholesterol can be targeted to lipid-laden foam cells in atherosclerotic plaques, helping to restore lipid homeostasis.

### 3.4 Modulating redox homeostasis

Maintaining redox balance is critical for cellular signaling and preventing oxidative damage. Artificial organelles offer powerful tools to manipulate the production and scavenging of reactive oxygen species (ROS).

#### 3.4.1 Peroxisome function mimicry and antioxidant synergy

Artificial peroxisomes are designed to mimic the role of natural organelles in ROS metabolism. A key strategy involves coencapsulating ROS-producing enzymes (*e.g.*, glucose oxidase, GOx) and ROS-scavenging enzymes (*e.g.*, catalase, CAT) [65]. By precisely controlling the stoichiometry and spatial arrangement of these enzymes, often within microfluidically generated vesicles (Sec. 2.2.2), it is possible to create systems that generate therapeutic ROS levels (for cancer therapy) or efficiently neutralize pathological ROS surges (in inflammatory diseases) [22]. Furthermore, synergistic designs can integrate multiple antioxidant enzymes (*e.g.*, superoxide dismutase (SOD) and CAT) to create a multistep scavenging cascade that is more effective than any single enzyme alone.

#### 3.4.2 Intervention in redox signaling pathways

Instead of directly acting on ROS, advanced artificial organelles can modulate the endogenous antioxidant response pathways of cells. For example, systems can be designed to deliver transcription factors such as Nrf2 or small molecules that activate the Nrf2-ARE pathway, thereby upregulating a suite of protective antioxidant genes. This approach leverages and amplifies the cell’s own defense mechanisms [66].

#### 3.4.3 Innovative strategies and mechanisms for regulating redox-sensitive molecules

A frontier in this area is the development of intelligent artificial organelles that can precisely regulate the spatiotemporal dynamics of redox-sensitive signaling molecules (*e.g.*, ROS and nitric oxide (NO)).

The strategy and mechanism are often integrated within a single system: 1) Enzyme-Mimetic Feedback Loops: Systems such as Fenozyme, a ferritin-based nanozyme, exhibit pH-dependent catalase-like activity. In the acidic tumor microenvironment, they efficiently scavenge H_2_O_2_, alleviating hypoxia and protecting the codelivered photosensitizer, thus creating a self-reinforcing therapeutic loop [2]. Here, the innovation lies in creating a feedback-driven system where the artificial organelle senses its environment and adapts its function accordingly. 2) Precision signal generation and release: The artificial NO nanotractor is another example where a MoS_2_-based nanostructure generates therapeutic NO under near-infrared (NIR) light irradiation. The mechanism involves light-triggered catalysis, and the strategy is to achieve on-demand, localized release of a transient signaling molecule, minimizing systemic side effects [67].

### 3.5 Principle of functional integration: from single-task to multi-task systems

**Fig. 3:**
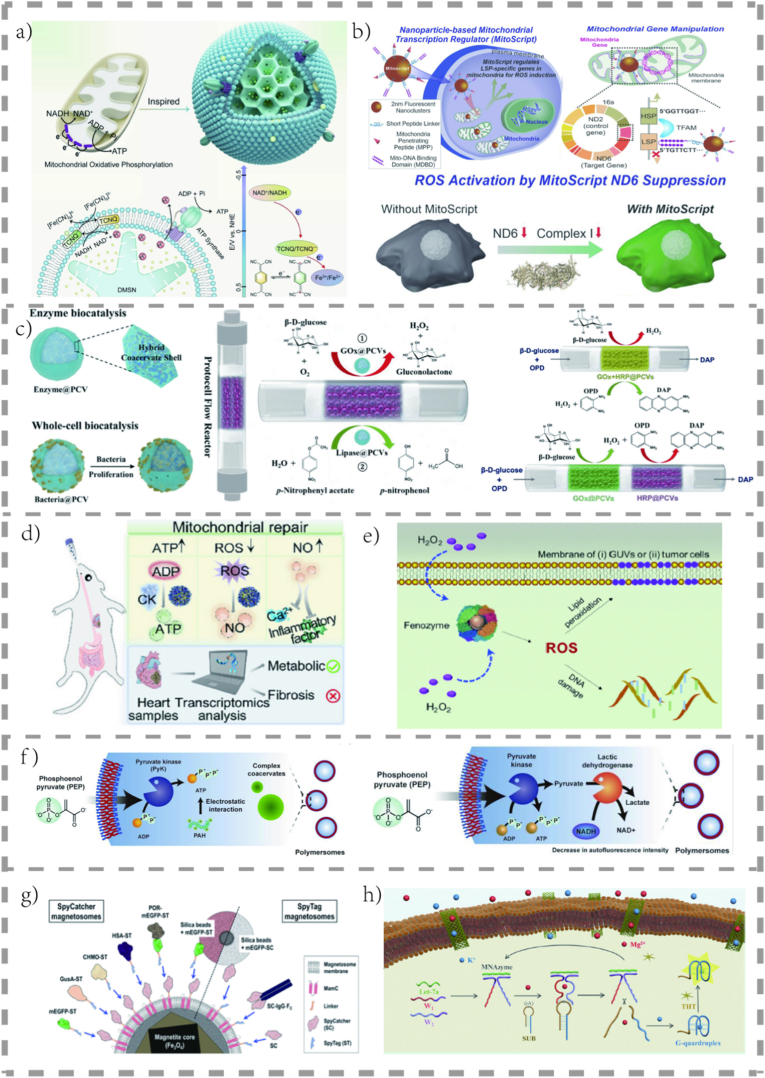
**Functional designs of artificial organelles for metabolic regulation.** a) An artificial mitochondrion for bioenergetic restoration. Schematic illustration of electron shuttling in an ATP synthase-reconstituted nanoarchitecture for enhanced bioenergetic anabolism, inspired by natural mitochondria. The cross section of this biolike system and transmembrane chemical reactions is presented below [3].© 2024, Angew Chem Int Ed Engl. b) A modular nanoparticle-based MitoScript platform for selective manipulation of mtDNA transcription, which provides a unique means of understanding and potentially treating mitochondria-associated diseases [54].© 2023, Nano Lett. c) Protocell continuous flow reactors consisting of a densely packed array of enzyme- or bacteria-containing membranized coacervate vesicles are constructed and used for the spatiotemporal processing of biochemical and whole-cell-mediated catalysis. Single-enzyme biocatalysis in PCV-based continuous-flow reactors. Multienzyme cascades using modulated PCV-based continuous flow reactors [56].© 2024, Adv Mater. d) Oral artificial mitochondrial nanorobots (AMNs) can treat ischemic heart disease by delivering ATP to damaged cardiomyocytes, modulating oxidative stress, and restoring cell viability. It improves energy metabolism and mitochondrial structure and reduces inflammation at the genetic level [51].© 2025, Adv Mater. e) A ferritin-based artificial peroxidase (nanozyme). A de novo artificial nanozyme composed of a ferritin heavy-chain scaffold protein and catalytic Fe_3_O_4_ nanoparticles as the active center mimics native peroxidase by utilizing Fenton catalysis reactions to generate free radicals [22].© 2021, American Chemical Society. f) Schematic illustration of the enzymatic reaction-induced complex coacervate formation in polymersomes. Schematic illustration of the PEP-driven cascade enzymatic reaction in polymersomes by incorporating both pyruvate kinase (PyK) and lactic dehydrogenase (LDH) [40].© 2022, Nat Commun. g) A flexible platform for targeted nanoassembly of multifunctional artificial glycocalyxes (MAGs) using the SpyTag-SpyCatcher (ST-SC) bioconjugate system, combining rapid chemical coupling with selective and controllable *in vivo* functionalization [24].© 2024, American Chemical Society. h) The ArtifCell@CNT system serves as a protective shield for signal probes, and the embedded CNTs facilitate probe transfer, exchange, and fusion between cells, thus achieving the effective sensing and monitoring of intracellular let-7a miRNA in living cells [46].© 2025, Adv Mater.

A recurring theme in the design of advanced artificial organelles is the transition from single-function modules to integrated, multitasking systems. This involves combining the functionalities described above. For example, an “oncometabolic regulator” can simultaneously inhibit glycolysis (Sec. 3.2.2), generate cytotoxic ROS (Sec. 3.4.1), and deliver a chemotherapeutic drug. This multipronged approach mirrors the complexity of disease pathology and is crucial for overcoming therapeutic resistance. The ultimate goal is to evolve from static, preprogrammed structures to dynamic, intelligent systems that can sense the cellular state (*e.g.*, through biosensors) and autonomously adjust their metabolic output, truly emulating the adaptive nature of natural organelles. The representative organelles in Chapter 3 are shown in Fig. 3.

## 4 Precision therapeutic applications for diseases

Building upon the functional designs detailed in Chapter 3, this chapter transitions from functional modules to disease-specific applications, highlighting how each engineered system can be harnessed to address specific pathological contexts. The central theme is precision intervention: disease-associated metabolic vulnerabilities are directly matched with artificial organelles designed to restore the corresponding functions. Specifically, dysfunctions in energy metabolism—such as impaired mitochondrial respiration or dysregulated glycolysis—are targeted by mitochondrial mimics (Sec. 3.2.1) and glycolysis regulators (Sec. 3.2.2); disturbances in biosynthesis and transport are countered by ER- and lipid-regulating systems (Sec. 3.3); and redox imbalance is mitigated by peroxisome-inspired constructs (Sec. 3.4). In the following sections, for each major disease, we first delineate the core metabolic dysregulation and then illustrate how rationally designed organelle systems—grounded in the construction strategies of Chapter 2 and the functional designs of Chapter 3—achieve not only symptomatic relief but also the restoration of metabolic homeostasis.

### 4.1 Neurodegenerative diseases: restoring neuronal metabolic health

A defining feature of neurodegenerative disorders such as AD and PD is neuronal metabolic failure, characterized by mitochondrial dysfunction, ATP depletion, and oxidative stress [68]. These metabolic defects trigger protein misfolding, neuroinflammation, and progressive neuronal loss. Artificial organelles provide a synthetic means to restore intracellular homeostasis through energy supplementation and ROS detoxification [69].

Most neurodegenerative phenotypes converge on two vulnerabilities: impaired oxidative phosphorylation and excessive ROS accumulation [70]. To address these, AO platforms integrate mitochondria-mimetic energy modules and peroxisome-like antioxidant modules, often in the same construct [3,71].

For instance, *Wang* et al. engineered human neural-stem-cell–derived artificial organelles that improved ATP synthesis and decreased mitochondrial ROS, enhancing oxidative phosphorylation efficiency *in vitro* and *in vivo* [72]. Likewise, *Kwon* et al. developed ceria-based nanozymes targeting mitochondria that decreased ROS levels by ∼60 % and rescued Aβ-induced cytotoxicity in SH-SY5Y neurons [73].

In the context of AD, artificial organelles have been engineered to specifically target β-amyloid (Aβ) plaques [74]. One representative strategy employs ceria-based nanozymes functionalized with Aβ-binding peptides, which enable selective accumulation at plaque sites and provide potent antioxidant activity, thereby alleviating local neurotoxicity [75]. This illustrates a key design principle: combining a metabolic function (redox modulation) with disease-specific targeting.

However, BBB penetration remains the critical translational bottleneck. Although mitochondria‐targeting groups such as triphenylphosphonium (TPP) enable precise subcellular localization, their use does not ensure brain delivery. Quantitative analyses indicate that systemically administered nanoparticles generally achieve only ∼0.1 % ID/g brain accumulation, even with optimized surface engineering (<1 %) [76]. Therefore, receptor-mediated or intranasal delivery routes are required for meaningful CNS exposur [77]. Moreover, inorganic nanomaterials may persist in brain tissue and activate microglia, underscoring the need for biodegradable or self-clearing carriers [78].

### 4.2 Cancer therapy: exploiting metabolic vulnerabilities

Cancer exemplifies a disease state where extensive metabolic reprogramming creates distinct opportunities for intervention [79]. Hallmarks such as the Warburg effect, disrupted redox homeostasis, and altered nutrient utilization expose metabolic nodes that can be precisely targeted [80]. Artificial organelles, designed to match these vulnerabilities, enable interventions that function selectively within the tumor microenvironment, achieving both therapeutic efficacy and contextual specificity.

#### 4.2.1 Starvation and metabolic disruption therapy

This strategy aims to cut off the energy supply of cancer cells. Drawing upon the principles of glycolysis modulation (Sec. 3.2.2), artificial organelles encapsulating glucose oxidase (GOx) can be delivered to the tumor microenvironment (TME). Gox-loaded AOs function as “metabolic traps,” consuming glucose and producing cytotoxic H_2_O [81].

Building on this concept, combinatorial strategies that pair GOx-mediated glucose depletion with autophagy inhibition have been explored to enhance tumor suppression. In preclinical models, such approaches have been reported to increase tumor cell apoptosis and reduce intracellular ATP levels, indicating more effective metabolic stress within the tumor microenvironment [82].

Nevertheless, this strategy has potential limitations. The strong O_2_ consumption by GOx can aggravate intratumoral hypoxia, which stabilizes HIF-1α and promotes the expression of angiogenic factors such as VEGF, potentially facilitating tumor invasion and metastasis [83]. Therefore, while GOx-based artificial organelles serve as effective metabolic traps, careful control of O_2_ depletion and combination with complementary interventions are necessary to maximize antitumor efficacy while minimizing hypoxia-induced adverse effects.

#### 4.2.2 Pro-oxidative “gas” therapy and synergistic approaches

Instead of fighting oxidation, this strategy amplifies it to kill cancer cells. Targeted ROS Generation: Systems are designed to generate high levels of ROS specifically within the TME [84]. For example, the PNCNzyme system, constructed via the self-assembly of polymers and the encapsulation of enzymes (a strategy related to that described in Sec. 2.1), exhibits pH-responsive peroxidase-like activity [85]. In the acidic TME, it catalyzes the conversion of a benign prodrug (indole-3-acetic acid, IAA) into toxic ROS, ensuring tumor-specific cytotoxicity [86].

Synergy with immunotherapy: Metabolic intervention can remodel the immunosuppressive TME. For example, by alleviating hypoxia (as a result of GOx-based therapy), artificial organelles can increase the efficacy of immune checkpoint inhibitors [87,88]. This highlights the shift toward multimodal therapies where metabolic regulation primes the tumor for other treatments [89,90]. Instead of scavenging ROS, gas therapy amplifies oxidative pressure specifically in the TME.

### 4.3 Cardiovascular diseases: correcting endothelial and myocardial metabolism

#### 4.3.1 Atherosclerosis intervention

Atherosclerosis arises from a complex interplay of lipid deposition, oxidative stress, and chronic inflammation within the arterial wall. AOs have been conceptualized as “nanocleaners” that selectively accumulate in atherosclerotic plaques, where they execute catalytic or anti-inflammatory functions analogous to endogenous hepatic detoxification systems [91].

These constructs are typically engineered to degrade cholesterol esters or neutralize ROS, thereby rebalancing redox homeostasis and lipid metabolism in vascular tissues. Among representative examples, macrophage-targeted AOs encapsulating antioxidant enzymes (*e.g.*, catalase, superoxide dismutase) or cholesterol ester hydrolase have demonstrated significant therapeutic benefit in ApoE^−/−^ murine models, achieving ∼40–50 % reductions in plaque area and markedly suppressing local cytokine secretion (TNF-α, IL-1β) [92].

By mitigating macrophage lipid overload and oxidative inflammation, these systems not only retard plaque progression but also restore endothelial redox balance and metabolic function, highlighting a mechanistically guided route toward vascular regeneration.

#### 4.3.2 Myocardial Ischemia‒Reperfusion (I/R) injury

I/R injury represents an acute metabolic crisis characterized by a burst of reactive oxygen species (ROS) and mitochondrial dysfunction upon restoration of blood flow. The abrupt redox imbalance and ATP depletion trigger cardiomyocyte apoptosis and irreversible myocardial remodeling [93].

Artificial organelles (AOs) have thus been developed as dual-purpose metabolic protectors, capable of simultaneous ROS scavenging and energy replenishment. By codelivering ATP-generating modules and antioxidant enzymes, these systems restore bioenergetic stability while neutralizing the oxidative surge, leading to significant recovery of cardiac function after infarction [94,95].

A representative design integrates ROS scavengers with mitochondrial protectants—for example, JP4-039–loaded or Pt-based nanozyme systems—that reduce infarct size by up to 35–40 % and preserve left ventricular ejection fraction (LVEF) in murine I/R model [96,97].

Such dual-functional artificial organelle architectures outperform conventional single-modality antioxidants, as they simultaneously mitigate oxidative stress and sustain ATP synthesis via restored oxidative phosphorylation, thereby preventing post-ischemic mitochondrial collapse [98].

Importantly, therapeutic efficacy is highly time-sensitive—administration within 30 min of reperfusion provides maximal protection, whereas delayed dosing markedly diminishes benefit.

These approaches enable quantitative prediction of myocardial uptake, residence time, and clearance, paving the way for precision-timed interventions that align AO activation with the transient biochemical window of reperfusion.

### 4.4 Inflammatory diseases and immune modulation

Chronic inflammation, as observed in rheumatoid arthritis (RA) or sepsis, is sustained by the metabolic reprogramming of immune cells. Artificial organelles can intervene by directly targeting these metabolic hubs [99].

#### 4.4.1 Rheumatoid arthritis (RA)

In RA, inflammatory macrophages in the synovial fluid exhibit increased glycolysis. Artificial organelles have been developed to specifically target these macrophages and deliver glycolysis inhibitors [100]. By restraining glycolytic flux, these systems suppress cytokine overproduction and thus dampen the inflammatory cascade [101,102].

In RA, synovial macrophages undergo metabolic reprogramming, exhibiting enhanced glycolysis that drives excessive pro-inflammatory cytokine production. To counteract this, AOs have been engineered to specifically target these inflammatory macrophages and deliver glycolysis inhibitor [100]. By restraining glycolytic flux, these systems effectively suppress IL-6 and TNF-α secretion [103,104], thereby dampening the inflammatory cascade and limiting tissue damage.

Advanced dual-functional AO architectures further integrate mitochondrial ATP supplementation, ensuring that inhibition of glycolysis does not compromise cellular bioenergetic. This metabolic balancing is critical, as it mitigates collateral tissue injury while maintaining macrophage viability for homeostatic functions.

For repeated or chronic administration, addressing the accelerated blood clearance (ABC) phenomenon is essential, as it can reduce therapeutic efficacy over multiple dosing cycles [105]. Consequently, biodegradable and low-immunogenic carriers are preferred, enabling sustained activity without provoking systemic immune responses.

#### 4.4.2 Sepsis

**Fig. 4:**
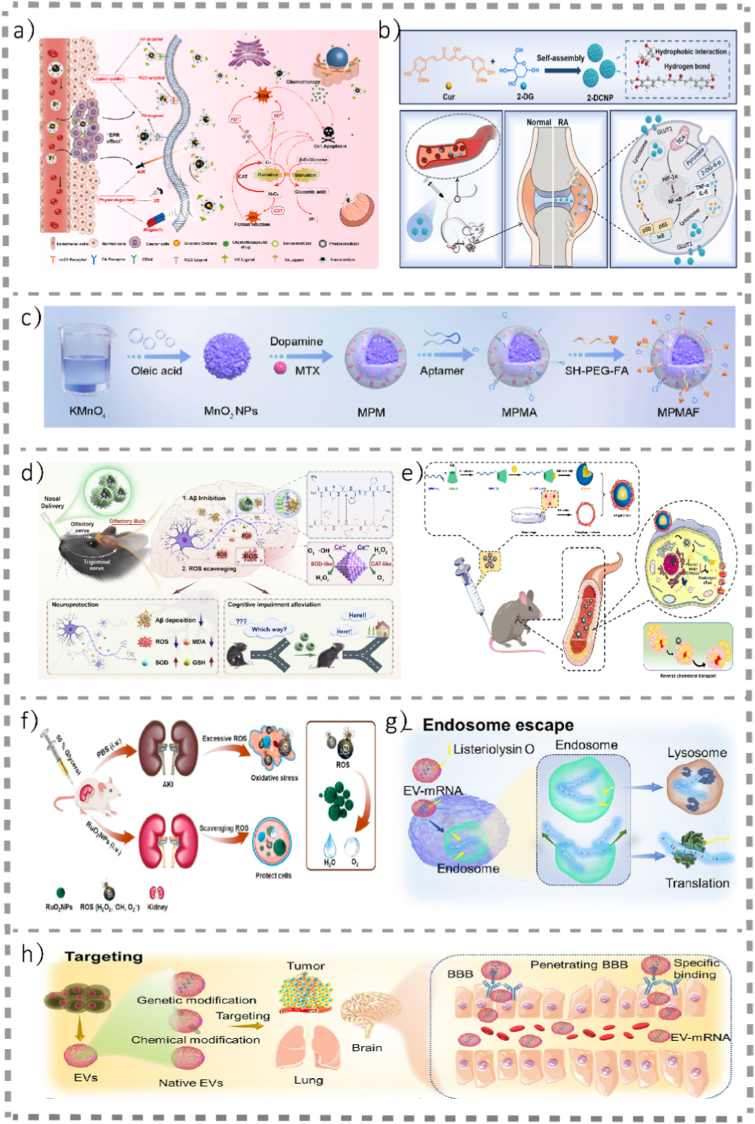
**Therapeutic applications of artificial organelles in major diseases** a) Schematic diagram of GOx-based nanocarriers for tumor-targeting therapy [90].©2023, Heliyon. b) Schematic illustration of the self-assembly process of 2-DG/Cur nanoparticles (2-DCNPs) and their in vivo behavior and application for RA treatment [101]. ©2024, Colloids Surf B Biointerfaces. c) Schematic of the design and preparation of MPMAF nanoplatforms [102]. ©2025, Small. d) KLVFF@LIP-CeO_2_ was intranasally delivered to APP/PS1 mice. KLVFF can inhibit Aβ aggregation via hydrogen-bonding interactions, whereas CeO_2_ can scavenge multiple types of ROS via facile redox switching between Ce^4+^ and Ce^3+^. This system can simultaneously alleviate Aβ deposition and oxidative stress in APP/PS1 mice, resulting in neuroprotective effects and the amelioration of cognitive impairment [75]. ©2024, Chemical Engineering Journal. e) Illustrations that MM@MTX NPs enhance reverse cholesterol transport in foam cells for the treatment of atherosclerosis [91]. ©2023, Journal of Nanobiotechnology. f) Schematic representation of the use of ultrasmall RuO_2_ NPs in ROS scavenging and acute kidney disease treatment [81].© 2020, American Chemical Society. g) Listeriolysin O, a special protein with the ability to accelerate endosomal membranes to form pores, is connected to EVs to increase the translation efficiency of mRNAs and endosome escape [109]. ©2025, Exploration. h) EVs derived from various cells have natural tumor-targeting effects. Genetic and nongenetic modifications further strengthen targeting, resulting in EVs targeting tumors, the brain, and the lung. EVs are modified with peptides, including RVG, iRGD, and c(RGDyK), to penetrate the BBB [109]. ©2025, Exploration.

Sepsis involves a systemic “cytokine storm” accompanied by excessive ROS generation [106]. Artificial organelles engineered with antioxidant capacity—such as peroxisome-mimetic systems—have shown promise in animal models by broadly scavenging ROS and mitigating multiorgan injury [107,108]. Therapeutic applications of artificial organelles in major diseases are detailed in Fig. 4. In order to guide readers in assessing performance of artificial organelles, the specific quantitative performance parameters of the representative artificial organelles mentioned in the above four paragraphs are shown in Table 4.

**Table 4:** Quantitative performance metrics of representative artificial organelles.

AO System/Name	Target Function	Key Performance Metric	Reported Value	Validation Level	Reference
ArtifCell@CNTs	miRNA Detection & Imaging	Detection Limit for let-7a miRNA	LOD: 31.2 fM	*In vitro & In vivo* (Living Cells)	Wu et al. Adv. Mater. 2025 [46]
i-Combisomes	Biomimetic Membrane & Vesicle Fusion	Lateral Diffusion Coefficient	D: 3–10 μm^2^/s	*In vitro*	Wagner et al. Adv. Sci. 2022 [26]
SC-MAGs	Enzyme Immobilization	Michaelis Constant (Km) of immobilized enzyme	Km: 0.29 mM	*In vitro*	Mickoleit et al. ACS Nano 2024 [24]
PFMACr AMNs	ATP Production	ATP Production vs. Natural Mitochondria	400 μg AMNs ≈1 × 10^7^ natural mitochondria (in 10^6^ injured cardiomyocytes)	*Invitro*	Li et al. Adv. Mater. 2025 [51]
Pluronic L121 Polymersomes	Spatiotemporal Enzymatic Reaction Control	Membrane Permeability Cut-off	Molecular Weight Cut-off: ∼500 Da	*Invitro*	Seo & Lee Nat. Commun. 2022 [40]
MitoScript	Mitochondrial Gene Regulation	Mitochondrial Targeting Efficiency	Co-localization with Mitochondria: ∼35 % (Pearson’s score)	*Invitro*	Yang et al. Nano Lett. 2023 [54]
MitoScript	ROS Level Modulation	ROS Induction via Gene Suppression	ROS Level Increase: ∼2.5-fold (vs. control, at 100 nM)	*Invitro*	Yang et al. Nano Lett. 2023 [54]
Mitochondria-Mimicking Nanoarchitecture (DMSM-NH_2_)	Bioenergy Anabolism (ATP Synthesis)	ATP Production Rate and Total ATP Production	13.7 nM/min,2.9 μM	*Invitro*	Wang et al. Angew. Chem. Int. Ed. 2024 [3]
DMSM-NH_2_	Proton Source Storage	NADH Load Capacity	735.7 mg/g	*Invitro*	Wang et al. Angew. Chem. Int. Ed. 2024 [3]
GUV-Fenozyme	Induction of Tumor Cell Death (DNA Damage)	DNA Content in Comet Tail (Comet Assay)	14.1 ± 6.9 % (at 200 μg/mL GUV-Fenozyme)	*In vitro (Cells)*	Hu et al. ACS Appl. Mater. Interfaces 2021 [22]
GUV-Fenozyme	Tumor Growth Inhibition	Bioluminescence Signal Reduction (Day 14)	Significant suppression vs. saline/GUV control groups	*In vivo (Mouse Model)*	Hu et al. ACS Appl. Mater. Interfaces 2021 [22]
CAMP-hMT1A Fusion Protein	Mitochondrial Antioxidant Delivery & ROS Scavenging	Restoration of intracellular ATP levels in PD cell model	ATP content: ∼200 % of MPP^+^-damaged cells (restored to near control levels)	*In vitro*	Kang et al. Exp Mol Med. 2018 [53]
Tyrosinase@PCV-CFR	Continuous-flow Biocatalysis & L-DOPA Synthesis	L-DOPA Production Rate	L-DOPA production: 1024 mg L^−1^ h^−1^ (at 50 μL min^−1^ flow rate)	*In vitro*	Ma et al.Adv Mater. 2024 [56]
MM@MTX NPs	Targeted cholesterol efflux & Anti-atherosclerosis Therapy	Plaque area reduction in ApoE^−/−^ mice	Atherosclerotic plaque area: 3.8 % (vs. 5.0 % for free MTX and 4.3 % for non-targeted MTX NPs)	*In vivo*	Zhu et al. J Nanobiotechnol 2023 [91]
SOD/CPO Nanogel	Intracellular ROS Conversion & Tumor Therapy	Tumor growth inhibition *in vivo*	Tumor suppression efficiency: ∼85 % (based on relative tumor volume)	*In vivo*	Wu et al. Nat Commun 2019 [110]
GOx/HRP Cascade Hydrogel	Tumor Starvation Therapy via Glucose Depletion	*In vitro* cell viability inhibition	Cell Viability (4T1 cells): <20 % after 24 h incubation	*In vitro*	Xu et al. ACS Mater. Lett. 2023 [111]
Fe[Gly]_2_/GOx Hydrogel	Ultrafast Cartilage Hydrogelation	Gelation time triggered by tissue fluid	Gelation Time: ∼5 s	*In vitro/Ex vivo*	Zhang et al. Angew. Chem. Int. Ed. 2021 [112]
GOx@PEG-MnFe_2_O_4_	Glucose depletion & magnetic hyperthermia	Specific Absorption Rate (SAR)	SAR: 296 W/g	*In vitro*	Anithkumar et al. ACS Appl.NanoMater. 2023 [81]
GOx@PEG-MnFe_2_O_4_	Glucose oxidase loading efficiency	Loading Efficiency	Loading Efficiency: ∼92.3 %	*In vitro*	Anithkumar et al. ACS Appl.Nano Mater. 2023 [81]
PtsaN-C	ROS scavenging (CAT-like activity)	Catalytic Efficiency (Km) and Maximum Reaction Velocity (Vmax)	Km: 19.33 mM (CAT-like), Vmax: 5.343 mg L^−1^·min^−1^ (CAT-like)	*In vitro*	Ye et al.Nat Commun 2024 [113]
2-DCNP	Glycolysis inhibition & anti-inflammatory therapy	Particle Size & Drug Loading	Size: 104.7 nm; DL: ∼100 %	*In vitro*	Chen et al. Colloids Surf B Biointerfaces 2024 [101]
2-DCNP	Arthritis symptom alleviation	Joint Score Reduction	Joint Score: Reduced from ∼3.5 to ∼1.0	*In vivo*	Chen et al. Colloids Surf B Biointerfaces 2024 [101]
MPMAF	Drug Delivery & Targeting	MTX Accumulation at RA Lesion	∼3.5-fold higher than free MTX	*In vivo*	Li et al. Small 2025 [102]
MPMAF	Drug Loading	MTX Loading Capacity	198.7 μg mL^−1^	*In vitro*	Li et al. Small 2025 [102]
MPMAF	ROS Scavenging	DPPH Radical Scavenging Rate	>80 % (at 50 μg mL^−1^)	*In vitro*	Li et al. Small 2025 [102]

## 5 Challenges and future perspectives

The journey from concept to clinical reality for artificial organelles is both exciting and fraught with challenges. While the preceding chapters have demonstrated remarkable progress in constructing (Chapter 2), programming (Chapter 3), and applying (Chapter 4) these biomimetic systems, significant hurdles remain. This final chapter will critically assess the key challenges impeding the field and outline the future perspectives that will guide the next generation of research, ultimately charting a course toward the realization of truly intelligent, life-like artificial organelles.

### 5.1 The critical bottlenecks: a tripartite challenge

#### 5.1.1 The scientific core: unresolved mechanistic complexity

Despite rapid advances in enzyme cascade construction and compartmental organization, the scientific foundation of AOs remains mechanistically opaque. Most existing studies focus on replicating specific catalytic or signaling functions—such as ROS scavenging or ATP regeneration—but overlook the emergent properties that arise from dynamic interactions between artificial and endogenous organelles [6,114]. True biological functionality is not defined by isolated reactions, but by reciprocal metabolic dialogues—the bidirectional exchange of metabolites, ions, and molecular cues that integrate subcellular behavior into systemic homeostasis [20].

A major obstacle is the lack of tools to monitor real-time communication between synthetic and native organelles. Current evaluation relies heavily on static microscopy, endpoint enzymatic assays, or bulk metabolomics, which fail to resolve transient fluxes, feedback inhibition, or redox coupling at the single-organelle level [115]. This limitation obscures the true mechanistic basis of therapeutic outcomes—whether metabolic improvements derive from the intended catalytic reaction or from secondary intracellular signaling cascades. Furthermore, most AO models operate in oversimplified *in vitro* environments devoid of cytoplasmic crowding, ionic gradients, or organelle–organelle cross-talk, leading to significant discrepancies when applied *in vivo* [116].

To bridge this gap, new integrative methodologies are needed—combining spatiotemporal metabolomics, single-organelle imaging, and in situ reaction flux tracking—to construct quantitative maps of AO–cell interactions. Computationally, systems-level metabolic modeling and agent-based simulations can help predict emergent behaviors prior to experimentation, identifying key control nodes and feedback vulnerabilities. Deciphering these mechanisms of “metabolic dialogue” will be crucial for moving from functional mimicry toward predictive control of cellular behavior, establishing the foundation for rational metabolic engineering of therapeutic AOs [117].

#### 5.1.2 The engineering core: scalability, robustness, and long-term trade-offs

From an engineering standpoint, reproducibility and scalability remain persistent obstacles. Current batch-based encapsulation methods yield inconsistent size distributions, enzyme loading, and catalytic activity, resulting in large performance variability *in vivo* [118]. While microfluidic continuous-flow synthesis and modular self-assembly have improved uniformity, these methods often reduce mechanical integrity and long-term catalytic retention [119]. Similarly, introducing inorganic or hybrid reinforcements (*e.g.*, MOF shells, silica matrices) enhances stability but restricts permeability and biocompatibility—illustrating a recurring trade-off between robustness and flexibility [19].

Quantitative benchmarking is still underdeveloped. Metrics such as polydispersity index (PDI), leakage ratio, and enzyme half-life (t_1_/_2_) should be standardized to compare AO systems across studies. Future designs may employ adaptive process control, where real-time feedback from microfluidic parameters is optimized via reinforcement learning algorithms to achieve consistent batch quality [120].

#### 5.1.3 The clinical core: immunogenicity and regulatory gaps

Immunogenicity and regulatory uncertainty form the most critical translational barriers. Although PEGylation remains the dominant “stealth” modification, it is associated with accelerated blood clearance (ABC) and anti-PEG antibody generation, which severely limit long-term tolerability [121]. Cell membrane–camouflaged AOs, particularly those engineered with CD47 or self-marker proteins, demonstrate superior immune evasion and circulation half-life [122]. However, their manufacturing complexity, batch variability, and potential interference with host immune signaling create formidable practical challenges [123].

Equally concerning is the absence of standardized immune-evaluation models and clear regulatory pathways for semi-living or self-adaptive nanotherapeutics. Quantifiable immunogenicity metrics—such as cytokine induction index or macrophage uptake ratio—are rarely reported, impeding systematic comparison [124]. Developing harmonized validation frameworks, akin to those used for cell or gene therapies, is essential to accelerate clinical translation while ensuring biosafety and reproducibility.

### 5.2 Future perspectives: toward intelligent, scalable, and clinically translatable artificial organelles

To overcome the inherent scientific, engineering, and clinical bottlenecks identified in Section 5.1, the future development of metabolism-based artificial organelles (AOs) must pivot from empirical design to a comprehensive, quantitative roadmap. This framework requires the synergistic integration of advanced computational tools, sophisticated multiscale engineering, and rigorous predictive modeling. Table 5 below summarizes the key challenge domains, aligning them with measurable quantitative metrics and emerging strategies, thus providing concrete parameters to guide translation from proof-of-concept to clinically relevant systems.

**Table 5:** Quantitative roadmap for overcoming key challenges in metabolism-based artificial organelle development.

Challenge Domain	Unresolved Key Problem	Measurable Metric (Example)	Current Solutions	Solution Strategy
Sci. Core	Multi-enzyme instability	Residual act. ≥70 % @24 h; ATP/ROS flux recovery	Enzyme encaps.; Cofactor regen.	AI-guided opt.; Biomimetic comp.
Eng. Core	Poor scalability & robustness	CV < 10 %; t_1_/_2_ ≥ 12 h	Batch opt.; Stabilizing polymers	Microfluidic continuous asm.; Self-healing mats.
Eng.–Sci. Hybrid	Low deep-tissue & sub-organel. targeting	Accum. ≥ 1–5 % ID	Peptide/Ab modif.	ML-screened ligands; Organelle-targeting moieties
Clin. Core (Safety)	Acute tox. & off-target eff.	Cytotox. < 10 %; enzyme ≤2 × baseline	Biocompat. scaffolds; Biodegr. carriers	Biomimetic cloak.; Predictive tox. model
Clin. Core (Safety)	Immunogenicity	IL-6, TNF-α; C3a, C5a	PEGyl.; Membrane coating	Gen. AI stealth design; ML epitope pred. *(2–5 y preclin.)*
Clin. Core (Transl.)	Lack of transl. evidence	≥2 species (≥1 large); 1st human trial	Std. preclin. workflow	AI/ML *in vivo* behav. pred.

#### 5.2.1 Toward “intelligent” and autonomous systems

A crucial trajectory involves developing AOs into intelligent and adaptive metabolic modules capable of sensing and autonomously responding to intracellular environments. This is achieved by embedding biosensors and dynamic feedback circuits directly within the AO architecture. Such systems can accurately monitor fluctuations in key metabolites, redox states, or pH, consequently triggering adaptive outputs like enzyme activation, optimized substrate channeling, or precise drug release [100]. This ability to maintain metabolic homeostasis under fluctuating conditions directly addresses the mechanistic complexity and cascade instability (Scientific Bottlenecks in Table 5).

Furthermore, longer-term strategies must pursue learning and evolutionary capabilities. Utilizing principles from directed evolution and computational optimization, AO populations could optimize their catalytic and interaction profiles in situ to maintain therapeutic efficacy across diverse, patient-specific metabolic landscape [125,126]. Given the inherent risks of off-target effects or uncontrolled autonomous behavior, the integration of rigorous safeguards and predictive computational safety modeling becomes paramount [127,128].

#### 5.2.2 Multiscale and multimodal integration

Addressing the engineering challenges of scalability, robustness, and functional longevity necessitates multiscale design strategies. These efforts must bridge nanoscale enzymatic activity with organ- or tissue-level function. Advanced fabrication techniques, particularly microfluidic assembly and 3D bioprinting, enable the spatial organization of distinct AO types—such as mitochondrial and peroxisomal mimics—into complex tissue-like metabolic bioreactor [129,130]. This hierarchical arrangement facilitates sustained and coordinated metabolic interventions in engineered tissues.

Moreover, integrating AOs with other therapeutic modalities dramatically enhances efficacy and systemic persistence. For example, AOs that supply ATP or neutralize reactive oxygen species can provide essential metabolic support for engineered immune cells (*e.g.*, CAR-T cells), significantly boosting cell survival and function within hostile tumor microenvironments [131].

#### 5.2.3 Embracing AI and computational design

The inherent design complexity of AOs makes AI and computational approaches indispensable for accelerating optimization and predicting complex biological performance. Machine learning models can be trained to predict organelle stability, cell interaction kinetics, and immune responses based on compositional and architectural parameters, thereby drastically reducing the time and resources required for experimental iteration [130,132].

Crucially, computational frameworks must transcend mere optimization to rigorously incorporate ethical and safety considerations [133]. By simulating potential off-target effects, metabolic rewiring, and systemic immunogenicity, these tools offer actionable insights for regulatory decision-making [134,135]. This approach directly addresses the clinical bottlenecks outlined in Table 5 by firmly linking quantitative assessment with translational readiness.

#### 5.2.4 Integrative outlook

**Fig. 5:**
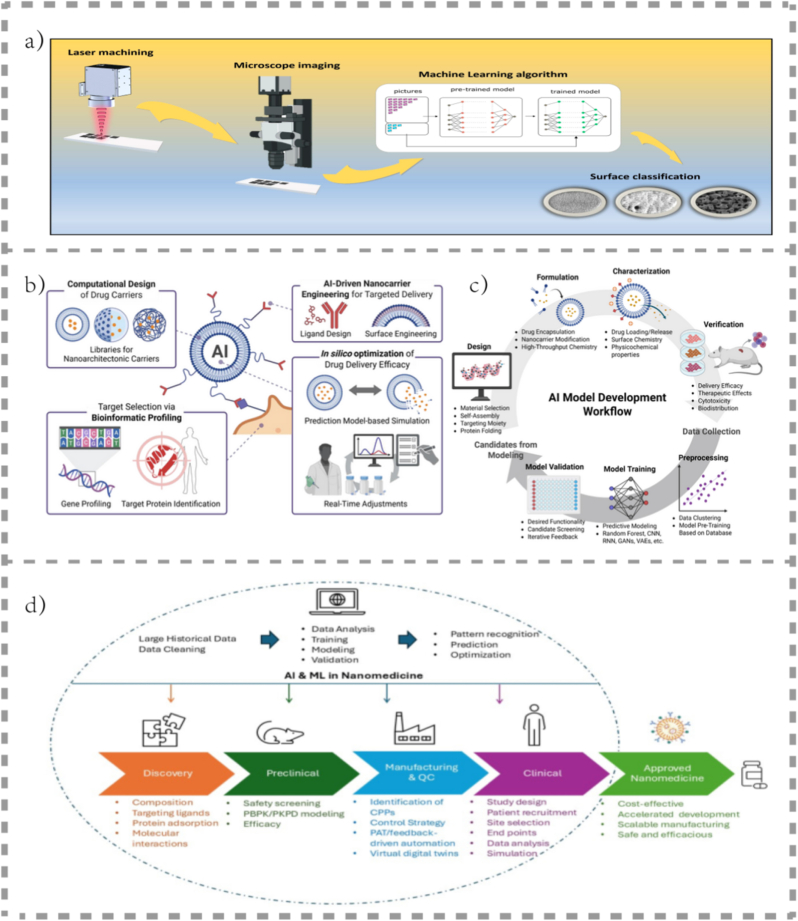
**Integration of artificial intelligence and nanoarchitectonics for intelligent and targeted drug delivery** a) Potential use of machine learning techniques in surface defect classification [136]. © MDPI 2024. b) Schematic representation of the integration between artificial intelligence (AI) and nanoarchitectonics for intelligent drug delivery [132].© 2025, Adv Mater. c) Schematic workflow of an AI algorithm for targeted drug delivery [132]. ©2025, Adv Mater. d) A graphical illustration showing the integration of AI and ML in different stages of nanomedicine product development [134]. ©2024, Pharm Res.

These converging directions define a closed-loop roadmap from mechanistic intelligence to engineering scalability and clinical predictability. The proposed framework emphasizes iterative “learn–build–validate” cycles: quantitative metrics guide design decisions, computational predictions accelerate testing, and experimental outcomes refine model parameters. By integrating adaptive feedback systems, multiscale engineering, and AI-guided design, metabolism-based AOs are poised to evolve from functional prototypes into adaptive, manufacturable, and robust therapeutic systems, marking a new frontier in synthetic medicine (More details are shown in Fig. 5).

## 6 Conclusion

The construction of artificial organelles represents a paradigm shift from traditional pharmacology to a new era of metabolic engineering and system reconstruction. By integrating principles from chemistry, materials science, synthetic biology, and medicine, researchers are no longer limited to simply inhibiting or activating single-molecule targets. Instead, a frontier of research is beginning to build de novo subcellular modules capable of executing complex, preprogrammed metabolic tasks with precision and intelligence. As the significant challenges in stability, biocompatibility, and intelligent control are overcome, these bioinspired machines hold the potential to revolutionize precision medicine. They offer a tangible path toward repairing, augmenting, and ultimately reprogramming cellular metabolism, promising transformative therapies for cancer, neurodegeneration, and other debilitating diseases. The grand challenge of reconstructing cellular life from the bottom up has begun, and artificial organelles are foundational building blocks.

## CRediT authorship contribution statement

**Keqiang Deng:** Writing – review & editing, Writing – original draft, Methodology. **Yihang Zhang:** Writing – original draft, Conceptualization. **Wenyu Jiang:** Data curation. **Yuxin Duan:** Methodology, Data curation. **Mei Gao:** Methodology, Investigation. **Min Zeng:** Software, Methodology, Formal analysis. **Jiehao Chen:** Visualization, Data curation. **Xiaoting Chen:** Visualization, Methodology. **Zhen Fan:** Methodology. **Chengli Yang:** Writing – review & editing, Supervision. **Kai Zhou:** Writing – review & editing, Writing – original draft, Supervision, Funding acquisition.

## Declaration of competing interest

The authors declare that they have no known competing financial interests or personal relationships that could have appeared to influence the work reported in this paper.

## Data Availability

No data was used for the research described in the article.
